# Testing Bidirectionality in Associations of Awareness of Age-Related Gains and Losses With Physical, Mental, and Cognitive Functioning Across 1 Year: The Role of Age

**DOI:** 10.1093/geronb/gbad150

**Published:** 2023-10-06

**Authors:** Serena Sabatini, Hans-Werner Wahl, Manfred Diehl, Linda Clare, Clive Ballard, Helen Brooker, Anne Corbett, Adam Hampshire, Blossom C M Stephan

**Affiliations:** School of Medicine, Institute of Mental Health, University of Nottingham, Nottingham, UK; Institute of Psychology, Heidelberg University, Heidelberg, Germany; Department of Human Development and Family Studies, Colorado State University, Forth Collins, Colorado, USA; Medical School, University of Exeter, Exeter, UK; Medical School, University of Exeter, Exeter, UK; Medical School, University of Exeter, Exeter, UK; Ecog Pro Ltd, Bristol, UK; Medical School, University of Exeter, Exeter, UK; Social, Genetic, and Developmental Psychiatry Centre, King’s College London, London, UK; School of Medicine, Institute of Mental Health, University of Nottingham, Nottingham, UK; Faculty of Health Sciences, Curtin enAble Institute, Curtin University, Bentley, Australia

**Keywords:** Causal pathway, Health, Mood, Self-perceptions of aging, Subjective aging

## Abstract

**Objectives:**

The bidirectionality between self-perceptions of aging and health-related outcomes may depend on age group. Therefore, we tested such bidirectionality among individuals in late midlife (50–64 years), young-old age (65-74 years), and old-old age (75+ years), taking advantage of the construct of Awareness of Age-Related Change (AARC) and its 2-dimensionality in terms of AARC-gains and AARC-losses. Various conceptualizations of physical, mental, and cognitive functioning were used as outcomes.

**Methods:**

Data from 2 measurement occasions (2019 and 2020) from the UK PROTECT study for individuals in late midlife (*N* = 2,385), young-old age (*N* = 2,430), and old-old age (*N* = 539) were used. Data on self-reported functional difficulties, depression, anxiety, and performance on four computerized cognitive tasks (i.e., verbal reasoning, paired associate learning, self-ordered search, and digit span) providing a score for verbal reasoning and a score for working memory were analyzed using cross-lagged panel models.

**Results:**

Across all 3 age groups, the bidirectional associations of AARC-gains with indicators of functioning were not significant, whereas higher AARC-losses significantly predicted slightly greater functional difficulties and higher depression and anxiety levels. Higher AARC-losses predicted slightly poorer Verbal Reasoning only in old-old age and poorer Working Memory predicted slightly higher AARC-losses only in young-old age. The remaining associations of AARC-losses with cognitive tasks were not statistically significant.

**Discussion:**

In accordance with previous research targeting other indicators of self-perceptions of aging, this study supported a stronger impact of AARC-losses on indicators of physical functioning and mental health than vice versa from midlife to old-old age.

There are many constructs capturing self-perceptions of aging (SPA). The most researched are subjective age and attitudes toward own aging. Subjective age captures how old individuals feel they are most of the time (Barrett, 2003). Attitudes toward own aging capture individuals’ perceptions and evaluations of the changes taking place in their lives while aging (Lawton, 1975). More positive SPA are associated with better physical, mental, and cognitive health, and with lower mortality risk (Levy et al., 2002; Li et al., 2021; Rippon & Steptoe, 2018; Seidler & Wolff, 2017; Spuling et al., 2013). One important limitation of the existing literature is that possible bidirectional associations between SPA and developmental outcomes have been largely unexplored. In Spuling et al. (2013) their 4-year longitudinal data supported a stronger predictive direction of subjective age on health outcomes, including subjective health, functional health, and depression, than the other way around. Further, an older subjective age predicted greater frailty (Li et al., 2021) and more functional difficulties (Rippon & Steptoe, 2018), whereas these physical indicators did not predict subjective age. Moreover, more negative attitudes toward own aging predicted greater cognitive decline over 20 years, but cognitive decline did not predict attitudes toward own aging (Siebert et al., 2020). However, Seidler and Wolff (2017) found evidence for a bidirectional relationship between SPA and cognitive decline. Overall, although some inconsistency has remained, the causal impact of SPA on health outcomes appears to be stronger than the reverse direction. A continuing gap in the literature is that nearly no findings exist on bidirectionality regarding multidimensional measures of SPA. This is unfortunate because there is a trend toward the increased use of multidimensional measures of SPA (e.g., Diehl & Wahl, 2010; Wurm et al., 2008).

The concept of Awareness of Age-Related Changes (AARC) and its corresponding questionnaire capture individuals’ perceptions of both their positive (AARC-gains) and negative (AARC-losses) changes across five life domains (Diehl & Wahl, 2010; Diehl et al., 2014), including cognitive functioning, socioemotional and sociocognitive functioning, physical health, interpersonal relations, and lifestyle and engagement. An example of an AARC-gain in the cognitive functioning domain is accumulated knowledge. An example of an AARC-loss in the physical health domain is reduced energy.

Cross-sectionally lower AARC-gains and higher AARC-losses are related to poorer scores on physical health indicators including self-rated health, lower functional ability, and more negative medical assessments (Sabatini, Silarova, et al., 2020). Higher AARC-gains and lower AARC-losses are also cross-sectionally related to better mood and psychological well-being (Sabatini, Silarova, et al., 2020; Sabatini, Ukoumunne, et al., 2020; Sabatini, Ukoumunne, Ballard, Collins, Corbett, et al., 2021). Moreover, higher AARC-losses show negligible to small cross-sectional associations with poorer subjective and objective cognitive performance (Sabatini, Ukoumunne, et al., 2020; Sabatini, Ukoumunne, Ballard, Collins, Anstey, et al., 2021; Zhu & Neupert, 2020). In fact, recent evidence suggests that AARC, as a multidimensional construct, is more strongly associated with health outcomes compared to unidimensional constructs (Sabatini, Ukoumunne, Ballard, Collins, Anstey, et al., 2021). In this study, we therefore used the AARC-gains and AARC-losses measures to examine bidirectionality.

## Physical, Mental, and Cognitive Functioning as Predictors of AARC-Gains and AARC-Losses

According to AARC theory, the experience of objective changes in a person’s physical, mental, and cognitive health and functioning can give rise to AARC-gains and AARC-losses experiences (Diehl & Wahl, 2010; Diehl et al., 2014). For example, those individuals who find it increasingly harder to remember things, experience new illnesses, or have low mood while aging may notice these negative changes in their daily life (Hughes & Touron, 2021) and, consequently, report lower AARC-gains and higher AARC-losses. So far, only one study (Sabatini, Siebert, et al., 2021) that used data for 103 German participants with mean baseline age of 62 years explored whether 20-year changes in cognitive, physical, and mental health predicted AARC-gains and AARC-losses. Results revealed that decline in cognitive functioning was a small predictor of fewer AARC-gains and more AARC-losses, whereas declines in physical and mental health were small-to-moderate predictors of more AARC-losses but not of fewer AARC-gains. More generally, the existing AARC literature suggests that, compared to lower perceived gains, higher perceived losses show stronger and more consistent associations with poorer developmental outcomes (Brothers et al., 2017; Dutt et al., 2016). A major explanation may lie in the stronger “negativity” of more negative age views on undermining outcomes as compared to stronger “positivity” of more positive age views on enhancing outcomes (Hummert, 2011; Meisner, 2012).

Further, although preliminary evidence supports the causal influence of health changes on AARC, there is also empirical evidence suggesting that individuals do not always represent change accurately (Lachman et al., 2008). For example, subjective and objective cognitive decline are not always related (Burmester et al., 2016). Additionally, individuals undergoing similar objective changes may differ in their subjective representation of such changes (Suchy et al., 2010).

## AARC-Gains and AARC-Losses as Predictors of Physical, Mental, and Cognitive Functioning

According to Stereotype Embodiment Theory (Levy, 2009), adults with positive perceptions of aging are more likely to maintain better health because they are more likely to adhere to a healthy lifestyle. In contrast, people who see losses as an inevitable part of aging and who perceive little or no control over their health are less likely to engage in healthy (e.g., physical exercise) and coping (e.g., seeking treatment for illnesses) behaviors (Levy et al., 2000). They also often experience adverse biological changes, such as cardiovascular stress (Levy et al., 2000). Consequently, people with negative perceptions of aging are at greater risk of developing chronic conditions and of mortality (Levy et al., 2002; Westerhof et al., 2023). Although it is likely that the AARC indicators behave similar in predicting health and functioning as other subjective aging indicators, respective empirical evidence is still limited.

Results of the few studies that have examined AARC as a predictor of physical and mental health support Levy’s Stereotype Embodiment Theory. For example, Brothers et al. (2020) found that in a sample of U.S. and German adults aged 40–98 years higher AARC-losses at baseline predicted poorer mental and physical health 2.5 years later and higher AARC-gains predicted better mental health 2.5 years later. Moreover, higher AARC-losses were found to predict more depressive symptoms up to 4.5 years later in German individuals aged 40–98 years (Dutt et al., 2018). Higher AARC-gains and lower AARC-losses also predicted better psychological well-being 12 months later in a sample of U.S. people aged 60 and older (Wilton-Harding & Windsor, 2022). In sum, longitudinal evidence of AARC on physical and mental health-related developmental outcomes including the examination of bidirectionality is limited. AARC-losses seemed to play a more important predictive role than AARC-gains. Also, there is currently no longitudinal evidence on AARC as a predictor of cognitive functioning.

## Emerging Evidence on Bidirectionality in AARC and Health Outcomes and the Role of Age Group

As hypothesized in the AARC theoretical framework (Diehl & Wahl, 2010; Diehl et al., 2014), it may be that individuals’ health and functioning influence individuals’ AARC-gains and AARC-losses which may, in turn, influence health and functioning through behavior (e.g., continued engagement in physical activity). So far, only one study has explored the bidirectional relationship between AARC and depression. It found that AARC-losses, but not AARC-gains, predicted change in depression, whereas 2-year change in depression did not predict change in AARC-losses (Dutt et al., 2016). Furthermore, the conceptualized bidirectional associations of AARC-gains and AARC-losses with indicators of physical and cognitive functioning have never been empirically tested.

Thus, we argue that testing the bidirectionality in the SPA and health connection needs to consider potentially different effects in different age groups. Objective and perceived age-related gains and losses; physical, mental, and cognitive functioning; and factors that influence both SPA and a person’s functioning (e.g., retirement, caring for aging parents, caring for children or grandchildren) differ greatly across late midlife, young-old, and old-old age (Baltes & Smith, 2003). More specifically, levels of AARC-losses consistently increase as people age (Brothers et al., 2016; Sabatini, Ukoumunne, et al., 2020; Testad et al., 2022; Wilton-Harding et al., 2022). Levels of AARC-gains also seem to differ across age groups although the direction of the association between AARC-gains and age is unclear. Whereas older individuals living in the United States, Germany, and Australia reported higher AARC-gains (Brothers et al., 2016; Wilton-Harding et al., 2022), older individuals living in the U.K. and Norway reported fewer AARC-gains (Sabatini, Ukoumunne, et al., 2020; Testad et al., 2022). Physical and cognitive health are also generally poorer among older people (World Health Organization, 2021). Regarding mental health indicators instead, depression seems to be less frequent in young-old age compared to late midlife, but seems to increase again in old-old age (Dooley & Kunik, 2017).

The size of the change experienced in both SPA and health over a given period of time may also differ across age groups. Indeed, in old-old age significant health changes typically occur over shorter timeframes compared to midlife and young-old age (McCarrey et al., 2016). Hence, because older people on average experience greater and more frequent objective health changes, the impact of health changes over SPA may be strongest in old-old age. At the same time, as age-related stereotypes and SPA become increasingly self-relevant with increasing age (Kornadt & Rothermund, 2012), the effect of AARC on future health may be greater in old-old age compared to midlife and young-old age (Siebert et al., 2020). So far, cross-sectional evidence suggests that the associations between mental health indicators and AARC-losses are stronger in old-old age compared to late midlife and young-old age (Sabatini, Ukoumunne, Ballard, Collins, Anstey, et al., 2021).

## The Current Study

This study used baseline and 1-year follow-up data from 5,354 individuals aged 50+ and enrolled in the UK PROTECT study. It investigated the strength and direction of the associations of AARC-gains and AARC-losses with indicators of functional status (instrumental activities of daily living [IADL]), mental health (depression; anxiety), and cognitive functioning (Verbal Reasoning; Working Memory). We expected to see additional support for the findings reported by previous studies that the direction of AARC predicting health outcomes is stronger compared to the reverse direction. However, as in the previous literature, compared to AARC-gains, AARC-losses have showed stronger impact on health outcomes (Brothers et al., 2017; Dutt et al., 2016), so we expected that this would be predominantly the case for AARC-losses.

Two additional issues were investigated in an exploratory fashion. First, given the mixed findings on AARC and cognitive performance, we refrained from stating a hypothesis. Second, we examined bidirectional associations across three groups of individuals in late midlife (50–64 years), young-old age (65–74 years), and old-old age (75+ years).

## Method

### Study Design

This study used data collected online through the UK PROTECT (Platform for Research Online to investigate Genetics and Cognition in Ageing) study (https://www.protectstudy.org.uk). Inclusion criteria were being a U.K. resident, English speaker, aged 50+, having internet access, and no clinical diagnosis of dementia at baseline. During recruitment, the UK PROTECT study was publicized by national advertisements that directed potential participants to the project website, where they could enroll in the study. UK PROTECT was also publicized among existing research cohorts of older adults including *Exeter 10,000*, *Join Dementia Research*, and *Brains for Dementia Research*. When joining the study, participants provided online informed consent at baseline. Ethics approval was obtained from the London Bridge NHS Research Ethics Committee and Health Research Authority (Ref:13/LO/1578).

In UK PROTECT, each year participants are invited via e-mail to take part in an online self-administered follow-up assessment, which participants can undertake via computer, smartphones, or tablets. For the purposes of this study, as part of their annual online assessments in January 2019 and 2020, participants were asked to complete additional optional questions assessing AARC. These were completed by 9,410 participants in 2019 (AARC assessment baseline in UK PROTECT) and 5,354 participants in 2020. The sample size was above the minimum number (*N* = 2,550) needed to detect a small effect of *r =* 0.01 with a power of 95%.

Supplementary Table 1 shows baseline descriptive statistics for the 5,354 participants who provided AARC data in 2020 and for the 4,056 participants who did not. Compared to those who did not provide data for the analyses due to withdrawal or no response on the selected questionnaires, the study sample included a significantly lower proportion of employed individuals and of individuals reporting poor health (1.8% in the study sample vs 2.8% of those excluded). Although the two samples differed in their age and levels of anxiety, depression, and cognitive functioning, differences were small in size. Less than 1% of participants self-reported a diagnosis of mild cognitive impairment.

### Measures

*Sociodemographic variables* comprised age, sex, ethnicity, and education (*secondary education, postsecondary education, vocational qualifications, undergraduate degrees, postgraduate degrees, and doctorates*).

*Awareness of Age-Related Change* was measured using the short form of the questionnaire (AARC-10 SF; Kaspar et al., 2019). This questionnaire has 10 items, with 5 items each assessing AARC-gains and AARC-losses, respectively. The AARC-10 SF does not allow for assessing the separate AARC behavioral domains, but rather produces scores for AARC-gains and AARC-losses across domains. However, each of the items of the AARC-gains and AARC-losses subscales represents one of the five AARC behavioral domains (Kaspar et al., 2019). Each item starts with the stem: “With my increasing age, I realize that…” Respondents rated how much each item applied to them on a 5-point Likert scale (1 = *not at all*; 2 = *a little bit*; 3 = *moderately*; 4 = *quite a bit*; 5 = *very much*). Scores were obtained for the AARC-gains and AARC-losses subscales by summing the five items within the respective subscales. Higher scores indicate higher AARC-gains/losses (range: 5-25). In this sample, Cronbach’s alpha for internal consistency for the AARC-gains subscale was .76 and for the AARC-losses subscale was .79.

*Functional Status* was assessed with Lawton’s IADL Scale (Lawton & Brody, 1969). Each of the seven items describes an activity of daily living, such as preparing meals. Respondents rated how difficult they found performing the activity (0 = *no difficulty*; 1 = *some difficulty*; 2 = *great difficulty*). Higher scores indicate greater functional difficulties (range: 0–14). In this sample, Cronbach’s α was 0.79.

### Mental Health

*Depression* was assessed with the nine-item Patient Health Questionnaire (Kroenke et al., 2001). For each item, respondents indicated how frequently they experienced the selected symptom over the previous 2 weeks (1 = *not at all*; 2 = *several days*; 3 = *more than half the days*; 4 = *nearly every day*). Higher scores (range: 9–36) indicate greater depression. Scores ≥19 indicate clinical depression. Cronbach’s α in this sample was 0.76.

*Anxiety* was assessed with the seven-item Generalized Anxiety Disorder scale (Spitzer et al., 2006). For each item, respondents indicated how frequently they experienced the selected symptom over the previous 2 weeks (1 = *not at all*; 2 = *several days*; 3 = *more than half the days*; 4 = *nearly every day*). Higher scores (range: 7–28) indicated greater presence of anxiety. Scores ≥12 indicate clinical anxiety. Cronbach’s α in this sample was 0.87.

### Cognitive Functioning

Cognitive functioning was measured with the PROTECT Cognitive Test Battery (Corbett et al., 2015), which is self-administered online and comprises four tasks: Verbal Reasoning, Paired Associate Learning, Self-Ordered Search, and Digit Span. For each task, a score was obtained by subtracting the number of errors from the number of correct answers; higher scores indicate better performance. For Verbal Reasoning the score has no upper or lower limit because respondents can make attempts on as many trials as they can within 3 min. For Paired Associate Learning, the score can range from 0 to 16. The score for the Self-Ordered Search Task can range from 0 to 20. For Digit Span, the score can range from 0 to 20. A composite score for Working Memory was obtained by summing participants’ scores on Paired Associate Learning, Self-Ordered Search, and Digit Span. This was deemed possible as intercorrelations among these variables were > .70. Higher scores on this linear composite indicate better Working Memory.

### Analyses

Cross-lagged panel models (see Supplementary Figure 1 for an example) were tested to examine the reciprocal relationships and directional influences that AARC-gains and AARC-losses have on indicators of functional status, mental health, and cognitive functioning over 1 year. Each model consisted of two predictors (baseline scores on either AARC-gains or AARC-losses and on the selected indicator of functional status, mental health, and/or cognitive functioning) and two outcomes (1-year follow-up scores on either AARC-gains or AARC-losses and on the selected indicator of functional status, mental health, and/or cognitive functioning). No latent variables were created for these models. AARC-gains and AARC-losses were allowed to correlate with the selected indicator of functional status, mental health, or cognitive functioning. Each model estimated two autoregressive paths, representing how stable scores on either AARC-gains or AARC-losses and on the selected indicator of functional status, mental health, or cognitive functioning were over 1 year. Each model also estimated two cross-lagged paths, representing the extent to which baseline AARC-gains and AARC-losses were associated with follow-up scores in the selected indicator of functional status, mental health, or cognitive functioning and the extent to which baseline scores in the selected indicator of functional status, mental health, or cognitive functioning were associated with follow-up levels of AARC-gains and AARC-losses.

Both unadjusted and adjusted cross-lagged panel models were estimated. Depending on the health indicator examined, models were adjusted for different covariates. The models investigating the bidirectional associations of AARC (gains and losses) with functional difficulties were adjusted for age, sex, depression, anxiety, Verbal Reasoning, and Working Memory at baseline because these variables are generally related with both AARC and functional difficulties (Gallo et al., 1997; Gure et al., 2013; Sabatini et al., 2022; Sabatini, Silarova, et al., 2020). The models investigating the bidirectional associations of AARC with depression and anxiety were adjusted for age, sex, functional difficulties, Verbal Reasoning, and Working Memory at baseline because these variables are typically related to mood and AARC (Gallo et al., 1997; Gure et al., 2013; Sabatini et al., 2022; Sabatini, Silarova, et al., 2020; Zlatar et al., 2018). Finally, the models investigating the bidirectional associations of AARC with Verbal Reasoning and Working Memory were adjusted for age, sex, functional difficulties, anxiety, and depression at baseline because these variables are generally related to AARC and cognitive functioning (Gure et al., 2013; Sabatini et al., 2022). We used a multigroup structural equation model approach to simultaneously fit the same cross-lagged panel model across the three age subsamples. To test whether the resulting coefficients differed across age subsamples, we used the Likelihood Ratio Test. The Likelihood Ratio Test was significant for each of the tested models, suggesting that coefficients were significantly different among age subsamples. This supported the subgroup approach at the analytical level.

Measures of functional status, mental health, and cognitive functioning were not completed by everyone as in UK PROTECT participants can decide which measures to complete. Because some of these measures were not completed by more than 2,651 participants in 2020, each cross-lagged panel model was conducted with the subsample of participants who completed the selected measure both at baseline and follow-up. Full Information Maximum Likelihood estimation was used to test the models. Analyses were conducted in STATA version 17.

## Results

### Descriptive Statistics

Descriptive statistics for study variables at baseline stratified by age group are presented in Supplementary Table 2. Out of the 5,354 study participants, 2,385 were in late midlife (50–64 years), 2,430 were in young-old age (65–74 years), and 539 were in old-old age (75+). Almost all participants were of White ethnicity. In all age groups, the majority of participants were women. Those in late midlife were better educated than those in young-old and old-old age.

Levels of AARC-gains were highest among those in late midlife and followed, in decreasing order, by those in young-old and old-old age, *F*-statistic (df) = 7.40 (2), *p* < .001. Levels of AARC-losses were lowest among those in late midlife and followed, in increasing order, by those in young-old age and old-old age, *F*-statistic (df) = 147.77 (2), *p* < .001. Although functional difficulties were low across all groups, those in old-old age reported about twice as many functional difficulties than those in late midlife and young-old age, *F*-statistic (df) = 7.81 (2), *p* < .001. On average, participants across all groups reported less depression and anxiety. However, those in late midlife reported higher depression, *F*-statistic (df) = 27.58 (2); *p* < .001, and anxiety scores, *F*-statistic (df) = 28.20 (2), *p* < .001 than those in young-old and old-old age. Scores on cognitive tasks were highest in late midlife and followed, in decreasing order, by those in young-old and old-old age, Verbal Reasoning *F*-statistic (df) = 118.54 (2), *p* < .001; Digit Span: *F*-statistic (df) = 28.09 (2), *p* < .001; Self-Ordered Search *F*-statistic (df) = 58.52 (2), *p* < .001; Paired Associate Learning F-statistic (df) = 65.45 (2), *p* < .001.

### Bidirectional Associations of AARC With Functional Status

Results from unadjusted and adjusted cross-lagged panel models testing the bidirectional influences of AARC-gains and AARC-losses with functional status are reported in Supplementary Table 3 and Table 1, respectively. In the adjusted models, in all age groups, AARC-gains and functional difficulties did not have a significant effect on each other. In late midlife and young-old age, AARC-losses and functional difficulties had a significant effect on each other, but AARC-losses had a stronger predictive effect on functional difficulties than the predictive effect exerted by functional difficulties on AARC-losses. In old-old age, AARC-losses had a significant effect on functional difficulties, but functional difficulties did not have a significant effect on AARC-losses. Hence, the causal direction from AARC to functional status was consistently stronger across age groups as compared to the reverse direction.

**Table 1.** Bidirectional Associations of AARC-Gains and AARC-Losses With Functional Difficulties by Age Group

Age group	AARC-gains				AARC-losses			
	Outcomes at follow-up	Predictors at baseline	β (95% CI)	*p* Value	Outcomes at follow-up	Predictors at baseline	β (95% CI)	*p* Value
Late midlife-adjusted models (*N* = 1,138)	AARC-gains	AARC-gains	.60 (.57, .62)	<.001	AARC-losses	AARC-losses	.68 (.66, .71)	<.001
		Functional difficulties	.01 (−.02, .04)	.511		Functional difficulties	.04 (.01, .06)	.011
	Functional difficulties	AARC-gains	−.02 (−.06, .02)	.438	Functional difficulties	AARC-losses	.21 (.16, .25)	<.001
		Functional difficulties	.50 (.47, .54)	<.001		Functional difficulties	.48 (.44, .51)	<.001
Young-old age-adjusted models (*N* = 1,244)	AARC-gains	AARC-gains	.59 (.57, .62)	<.001	AARC-losses	AARC-losses	.68 (.65, .70)	<.001
		Functional difficulties	−.02 (−.05, .01)	.176		Functional difficulties	.03 (.002, .06)	.038
	Functional difficulties	AARC-gains	−.01 (−.05, .03)	.572	Functional difficulties	AARC-losses	.12 (.07, .16)	<.001
		Functional difficulties	.43 (.39, .47)	<.001		Functional difficulties	.42 (.38, .46)	<.001
Old-old age-adjusted models (*N*= 269)	AARC-gains	AARC-gains	.64 (.59, .69)	<.001	AARC-losses	AARC-losses	.65 (.60, .70)	<.001
		Functional difficulties	−.01 (−.08, .05)	.667		Functional difficulties	.04 (−.02, .10)	.242
	Functional difficulties	AARC-gains	−.02 (−.12, .07)	.644	Functional difficulties	AARC-losses	.24 (.14, .34)	<.001
		Functional difficulties	.31 (.22, .41)	<.001		Functional difficulties	.28 (.18, .37)	<.001

*Notes*: AARC = awareness of age-related change; CI = confidence interval.

Models are adjusted for age, sex, anxiety at baseline, depression at baseline, verbal reasoning at baseline, and working memory at baseline.

### Bidirectional Associations of AARC With Mental Health

Results from unadjusted and adjusted cross-lagged panel models testing the bidirectional influences of AARC-gains and AARC-losses with depression are reported in Supplementary Table 4 and Table 2, respectively. In the adjusted models, AARC-gains and depression did not have a significant effect on each other in any age group. In contrast, AARC-losses and depression had a significant effect on each other across all age groups. Compared to the predictive effect of depression on AARC-losses, the predictive effect of AARC-losses on depression was consistently significantly stronger across all age groups.

**Table 2.** Bidirectional Associations of AARC-Gains and AARC-Losses With Depression by Age Group

Age group	AARC-gains				AARC-losses			
	Outcomes at follow-up	Predictors at baseline	β (95% CI)	*p* Value	Outcomes at follow-up	Predictors at baseline	β (95% CI)	*p* Value
Late midlife-adjusted models (*N* = 1,138)	AARC-gains	AARC-gains	.60 (.57, .62)	<.001	AARC-losses	AARC-losses	.68 (.66, .71)	<.001
		Functional difficulties	−.03 (−.06, .003)	.076		Functional difficulties	.10 (.07, .13)	<.001
	Functional difficulties	AARC-gains	−.01 (−.05, .03)	.626	Functional difficulties	AARC-losses	.26 (.22, .30)	<.001
		Functional difficulties	.52 (.48, .55)	<.001		Functional difficulties	.43 (.39, .47)	<.001
Young-old age-adjusted models (*N* = 1,244)	AARC-gains	AARC-gains	.59 (.57, .62)	<.001	AARC-losses	AARC-losses	.68 (.65, .70)	<.001
		Functional difficulties	−.01 (−.04, .03)	.747		Functional difficulties	.12 (.09, .15)	<.001
	Functional difficulties	AARC-gains	−.03 (−.07, .01)	.142	Functional difficulties	AARC-losses	.22 (.18, .26)	<.001
		Functional difficulties	.55 (.52, .59)	<.001		Functional difficulties	.49 (.45, .53)	<.001
Old-old age-adjusted models (*N* = 269)	AARC-gains	AARC-gains	.64 (.59, .69)	<.001	AARC-losses	AARC-losses	.65 (.60, .70)	<.001
		Functional difficulties	−.05 (−.12, .01)	.117		Functional difficulties	.07 (.01, .14)	.025
	Functional difficulties	AARC-gains	−.04 (−.12, .03)	.268	Functional difficulties	AARC-losses	.11 (.02, .19)	.018
		Functional difficulties	.70 (.64, .75)	<.001		Functional difficulties	.66 (.60, .73)	<.001

*Notes*: AARC = awareness of age-related change; CI = confidence interval.

Models are adjusted for age, sex, functional difficulties at baseline, verbal reasoning at baseline, and working memory at baseline.

Results from unadjusted and adjusted cross-lagged panel models testing the bidirectional influences of AARC-gains and AARC-losses with anxiety are reported in Supplementary Table 5 and Table 3, respectively. In the adjusted models, AARC-gains and anxiety did not have a significant effect on each other in any age group. AARC-losses and anxiety had a significant effect on each other in late midlife and young-old age. Compared to the effect of anxiety on AARC-losses, the effect of AARC-losses on anxiety was stronger in late midlife and young-old age. In old-old age, although AARC-losses had a significant effect on anxiety, anxiety did not have a significant effect on AARC-losses.

**Table 3.** Bidirectional Associations of AARC-Gains and AARC-Losses With Anxiety by Age Group

Age group	AARC-gains				AARC-losses			
	Outcomes at follow-up	Predictors at baseline	β (95% CI)	*p* Value	Outcomes at follow-up	Predictors at baseline	β (95% CI)	*p* Value
Late midlife-adjusted models (*N* = 1,138)	AARC-gains	AARC-gains	.60 (.57, .62)	<.001	AARC-losses	AARC-losses	.70 (.68, .72)	<.001
		Functional difficulties	−.02 (−.05, .01)	.243		Functional difficulties	.06 (.03, .09)	<.001
	Functional difficulties	AARC-gains	.02 (−.02, .07)	.340	Functional difficulties	AARC-losses	.19 (.14, .23)	<.001
		Functional difficulties	.49 (.45, .53)	<.001		Functional difficulties	.44 (.41, .48)	<.001
Young-old age-adjusted models (*N* = 1,244)	AARC-gains	AARC-gains	.59 (.57, .62)	<.001	AARC-losses	AARC-losses	.69 (.67, .71)	<.001
		Functional difficulties	.01 (−.02, .04)	.541		Functional difficulties	.09 (.06, .12)	<.001
	Functional difficulties	AARC-gains	−.02 (−.06, .03)	.433	Functional difficulties	AARC-losses	.17 (.13, .21)	<.001
		Functional difficulties	.46 (.42, .50)	<.001		Functional difficulties	.42 (.38, .46)	<.001
Old-old age-adjusted models (*N* = 269)	AARC-gains	AARC-gains	.64 (.59, .69)	<.001	AARC-losses	AARC-losses	.67 (.62, .72)	<.001
		Functional difficulties	−.02 (−.09, .04)	.516		Functional difficulties	.03 (−.03, .10)	.258
	Functional difficulties	AARC-gains	−.03 (−.12, .06)	.525	Functional difficulties	AARC-losses	.18 (.09, .27)	<.001
		Functional difficulties	.59 (.51, .66)	<.001		Functional difficulties	.54 (.46, .62)	<.001

*Notes*: AARC = awareness of age-related change; CI = confidence interval.

Models are adjusted for age, sex, functional difficulties at baseline, verbal reasoning at baseline, and working memory at baseline.

Hence, once again, the directional effects from AARC to depression and anxiety were consistently stronger across age groups as compared to the reverse direction.

### Bidirectional Associations of AARC With Cognitive Functioning

Results from unadjusted and adjusted cross-lagged panel models testing the bidirectional influences of AARC-gains and AARC-losses with Verbal Reasoning are reported in Supplementary Table 6 and Table 4, respectively. In the adjusted models, in late midlife, young-old age, and old-old age AARC-gains and Verbal Reasoning did not have a significant effect on each other. In late midlife and young-old age, AARC-losses and Verbal Reasoning did not have a significant effect on each other, whereas in old-old age higher AARC-losses had a significant negative effect on Verbal Reasoning, but Verbal Reasoning did not have a significant effect on AARC-losses.

**Table 4.** Bidirectional Associations of AARC-Gains and AARC-Losses With Scores on Verbal Reasoning by Age Group

Age group	AARC-gains				AARC-losses			
	Outcomes at follow-up	Predictors at baseline	β (95% CI)	*p* Value	Outcomes at follow-up	Predictors at baseline	β (95% CI)	*p* Value
Late midlife-adjusted models (*N* = 1,138)	AARC-gains	AARC-gains	.60 (.57, .63)	<.001	AARC-losses	AARC-losses	.72 (.69, .74)	<.001
		Functional difficulties	−.01 (−.05, .03)	.595		Functional difficulties	−.02 (−.05, .01)	.258
	Functional difficulties	AARC-gains	−.03 (−.06, .01)	.116	Functional difficulties	AARC-losses	−.001 (−.03, .03)	.965
		Functional difficulties	.74 (.71, .76)	<.001		Functional difficulties	.74 (.71, .76)	<.001
Young-old age-adjusted models (*N* = 1,244)	AARC-gains	AARC-gains	.59 (.57, .62)	<.001	AARC-losses	AARC-losses	.71 (.69, .73)	<.001
		Functional difficulties	−.03 (−.06, .01)	.147		Functional difficulties	−.02 (−.05, .01)	.232
	Functional difficulties	AARC-gains	−.01 (−.04, .03)	.773	Functional difficulties	AARC-losses	−.03 (−.06, .01)	.128
		Functional difficulties	.74 (.72, .77)	<.001		Functional difficulties	.74 (.72, .77)	<.001
Old-old age-adjusted models (*N* = 269)	AARC-gains	AARC-gains	.64 (.59, .69)	<.001	AARC-losses	AARC-losses	.68 (.63, .72)	<.001
		Functional difficulties	−.02 (−.09, .06)	.639		Functional difficulties	.04 (−.03, .10)	.292
	Functional difficulties	AARC-gains	−.02 (−.09, .05)	.653	Functional difficulties	AARC-losses	−.10 (−.17, −.02)	.010
		Functional difficulties	.75 (.70, .80)	<.001		Functional difficulties	.75 (.70, .80)	<.001

*Notes*: AARC = awareness of age-related change; CI = confidence interval.

Models are adjusted for age, sex, functional difficulties at baseline, anxiety at baseline, and depression at baseline.

Results from unadjusted and adjusted cross-lagged panel models testing the bidirectional influences of AARC-gains and AARC-losses with Working Memory are reported in Supplementary Table 7 and Table 5, respectively. In the adjusted models for late midlife, AARC-gains did not have a significant effect on Working Memory, whereas poorer Working Memory had a significant effect on AARC-gains. Furthermore, AARC-losses and Working Memory did not have a significant effect on each other. In young-old age, AARC-gains and Working Memory did not have a significant effect on each other. In young-old age, poorer Working Memory predicted more AARC-losses, but AARC-losses did not predict Working Memory. In old-old age, AARC (gains and losses) and Working Memory did not significantly predict each other.

**Table 5.** Bidirectional Associations of AARC-Gains and AARC-Losses With Scores on Working Memory by Age Group

Age group	AARC-gains				AARC-losses			
	Outcomes at follow-up	Predictors at baseline	β (95% CI)	*p* Value	Outcomes at follow-up	Predictors at baseline	β (95% CI)	*p* Value
Late midlife-adjusted models (*N* = 1,138)	AARC-gains	AARC-gains	.60 (.57, .62)	<.001	AARC-losses	AARC-losses	.68 (.66, .71)	<.001
		Functional difficulties	−.05 (−.08, −.01)	.016		Functional difficulties	−.01 (−.04, .02)	.513
	Functional difficulties	AARC-gains	−.03 (−.07, .01)	.126	Functional difficulties	AARC-losses	.001 (−.04, .05)	.965
		Functional difficulties	.57 (−.07, .01)	<.001		Functional difficulties	.57 (.53, .61)	<.001
Young-old age-adjusted models (*N* = 1,244)	AARC-gains	AARC-gains	.60 (.57, .62)	<.001	AARC-losses	AARC-losses	.68 (.65, .70)	<.001
		Functional difficulties	.01 (−.02, .05)	.540		Functional difficulties	−.05 (−.08, −.02)	.002
	Functional difficulties	AARC-gains	−.04 (−.08, .001)	.056	Functional difficulties	AARC-losses	−.01 (−.05, .04)	.763
		Functional difficulties	.56 (.52, .59)	<.001		Functional difficulties	.55 (.51, .59)	<.001
Old-old age-adjusted models (*N* = 269)	AARC-gains	AARC-gains	.64 (.59, .69)	<.001	AARC-losses	AARC-losses	.65 (.60, .70)	<.001
		Functional difficulties	−.02 (−.10, .05)	.560		Functional difficulties	.01 (−.06, .08)	.779
	Functional difficulties	AARC-gains	−.02 (−.11, .07)	.596	Functional difficulties	AARC-losses	−.03 (−.13, .07)	.601
		Functional difficulties	.55 (.46, .63)	<.001		Functional difficulties	.55 (.47, .64)	<.001

*Notes*: AARC = awareness of age-related change; CI = confidence interval.

Models are adjusted for age, sex, functional difficulties at baseline, anxiety at baseline, and depression at baseline.

In sum, in contrast to physical status and mental health, evidence on bidirectionality between AARC and indicators of cognitive functioning was mixed and inconsistent.

## Discussion

This study investigated, among individuals in late midlife, young-old age, and old-old age, the bidirectional associations of AARC-gains and AARC-losses with indicators of functional status, mental health, and cognitive functioning. AARC-losses had a stronger predictive effect on functional difficulties, depression, and anxiety compared to the effects of these variables on AARC-losses. Higher AARC-losses had a small effect on poorer Verbal Reasoning only in old-old age and Working Memory had a small effect on AARC-losses only in young-old age. The remaining associations of AARC-losses with Verbal Reasoning and Working Memory were not statistically significant. The bidirectional associations of AARC-gains with indicators of functional status, mental health, and cognitive functioning were statistically not significant. Overall, results support Stereotype Embodiment Theory (Levy, 2009) stating that negative SPA have a detrimental impact on a person’s future health and functioning. According to Levy’s theory, individuals with negative SPA have internalized ageist stereotypes, and the application of these negative stereotypes to themselves makes them less likely to engage in health-enhancing behaviors such as physical activity (Klusmann et al., 2012). Investigation of the 1-year bidirectional influences between AARC and health-enhancing behaviors, such as physical activity and social and cultural engagement, in the current study sample has been reported elsewhere. This work found that higher AARC-losses predicted less engagement in physical, social, and cultural activities at the cross-sectional level and 1 year later.

In this 1-year study, AARC-losses and cognitive functioning did not influence each other in late midlife. In young-old age, Working Memory had an effect on AARC-losses and in old-old age AARC-losses had an effect on Verbal Reasoning. This pattern of results may suggest that although AARC-losses exert a significant effect on mental health and functional status over a 1-year period across all age groups, the potential effect of AARC-losses on cognitive functioning may be exerted over a longer period, especially in late midlife and young-old age. In contrast, in old-old age a period of 1 year may be sufficient to detect significant change in cognitive functioning. Nonetheless, a 1-year follow-up may be a limited period also for the old-old individuals who enrolled and stayed in the UK PROTECT cohort due to selection and retention bias. In support of the need for studies with longer follow-ups, Seidler and Wolff (2017) found that over 3 years subjective perceptions of aging of individuals aged 40–93 years can indeed influence cognitive decline and vice versa. Moreover, Siebert et al. (2020) showed that attitudes toward own aging predicted 20-year cognitive change among individuals in their 60s but not among individuals in their 40s.

The findings that AARC-losses exerted a greater effect on functional difficulties extend results of other SPA concepts, such as subjective age (e.g., Rippon & Steptoe, 2018), to the AARC construct. Moreover, in our study, AARC-losses had a stronger predictive effect on depression compared to the predictive effect of depression on AARC-losses, and AARC-gains and depression did not predict each other. This pattern of results also replicates results by Dutt et al. (2016) and by Brothers et al. (2020) in a larger sample of U.K. individuals and across subgroups of people in late midlife, young-old age, and old-old age. Moreover, in our study, we extended these results to anxiety for the first time, suggesting that more negative SPA may have a broader negative impact on future mental health.

Given the effect that negative AARC-losses had on mental health and functional status in a short period of 1 year and given that negative perceptions of aging pervade western societies, reducing negative perceptions of aging may be a useful means to promote healthy aging (Levy, 2017). In the United States, health conditions due to the consequences of negative perceptions of aging are estimated to cost $33.7 billion each year (Levy et al., 2020). Recent evidence even found that, among people aged ≥80 years, those with lower AARC-gains and higher AARC-losses were at greater risk of mortality 3.5 years later (Kaspar et al., 2021), further highlighting the importance of promoting positive SPA.

At the societal level, organizations representing older adults need to continue their action to reduce and eventually eradicate erroneous perceptions of aging and to promote inclusion of older adults within society (see Diehl et al., 2020). An example of action that could be undertaken toward this aim would be national campaigns promoting better understanding of the contribution that older people make within society and realistic knowledge about the changes associated with aging (see Diehl et al., 2020, for suggestions). Several local (e.g., Age UK) and international (e.g., World Health Organization) organizations are already working in this direction. Similarly, in the United States, several organizations have initiated the Reframing Aging initiative (www.reframingaging.org) to address societal ageism and implicit bias against older adults by reframing the current negative narrative on aging (see Diehl et al., 2020). Because perceptions of aging start to develop in childhood (Levy, 2017), another early intervention strategy promoting positive perceptions of aging is the delivery of “awareness events” in schools aiming to sensitize children and youth to the topic of aging. Increasing knowledge of age-related changes and decreasing stigma associated with aging in the general population may promote better integration of older people within society and consequently foster their health and well-being.

### Strengths and Limitations

This was the first study exploring the bidirectional associations of AARC-gains and AARC-losses with functional difficulties and cognitive functioning. This was among the first studies linking AARC to anxiety symptoms. Other strengths of this study include the large sample, the use of several indicators per domain of functioning, and stratification of analyses by age groups. Stratifying the analyses by age groups was important given the different developmental and health-related changes that people in late midlife, young-old age, and old-old age typically face. However, it is possible that due to selection bias and survivor effect—that is due to healthier old-old people having enrolled and stayed in the study—old-old people in the current study do not well represent the health status and age-related perceptions of the wider population of old-old individuals.

This study has other limitations, too. First, although the length of the follow-up was long enough to detect significant associations between AARC and functional status and mental health, 1 year may have not been long enough to detect associations between AARC and changes in cognitive functioning, especially in late midlife and young-old age when cognitive decline is not a normative experience yet (e.g., Schaie, 2013). Second, a significant proportion of participants was lost to follow-up. However, differences between the study sample and excluded participants were very small at baseline. Third, a limitation of this study is that functional status was not assessed with objective measures. Fourth, the sample comprised a majority of women that overrepresent the proportion of women in the U.K. population. This is a limitation as women tend to maintain better health in older age than men. Fifth, the sample comprised individuals with above-average education and self-rated health, with access to the internet, and who used technologies such as smartphones, computers, and tablets. Hence, generalizability of study results to the wider population, including individuals who are less educated, are less healthy, and are not technology literates, may be limited by a cohort bias. Future studies should investigate whether in more heterogeneous samples in terms of sociodemographic characteristics and health status, the associations between AARC-losses and health indicators are stronger. Sixth, in this study, we assessed working memory and verbal reasoning, but other cognitive abilities, such as general knowledge and executive functions, were not assessed and may show different bidirectional patterns with AARC.

## Conclusions

Over a 1-year period, greater perceptions of age-related losses had a significant negative effect on the functional status and mental health of U.K. adults. Moreover, this effect was consistent among individuals in late midlife, young-old age, and old-old age. AARC-losses also had a small negative impact on the verbal reasoning abilities of those in old-old age. Results highlight the opportunity that the promotion of more positive perceptions of aging may offer to promote healthy aging.

## Supplementary Material

gbad150_suppl_Supplementary_Tables_S1-S7_Figures_S1

## Data Availability

UK PROTECT data are available upon request and approval from the UK PROTECT steering committee.

## Preregistration

Research questions for this study were presented to and approved by the PROTECT steering committee. Research questions were also submitted as a research proposal for an ESRC Postdoctoral Fellowship, which has been awarded to the first author. Positive perceptions of aging could be promoted both at the individual and societal levels. At the individual level, several intervention programs promoting positive SPA, including increasing age-related perceptions of gains and decreasing age-related perceptions of losses, have been developed. They all have shown that promoting positive SPA is possible and leads to more active and healthy aging (Diehl et al., 2022; Knight et al., 2021; Nehrkorn-Bailey et al., 2023). The promotion of positive SPA should, however, be done in a sensitive and realistic way because, compared to younger adults, older adults may have more age-related perceptions of losses that accurately reflect their personal experiences (e.g., poorer objective health). Nonetheless, it is important to convey to middle-aged and older adults that they can have a certain amount of control over their aging, and this may increase positive SPA.
