# Supplementary material for: Testing Bidirectionality in Associations of Awareness of Age-Related Gains and Losses With Physical, Mental, and Cognitive Functioning Across 1 Year: The Role of Age
Source: J Gerontol B Psychol Sci Soc Sci. 2023 Oct 6;78(12):2026–36. doi: 10.1093/geronb/gbad150 (PMC10699739; doi:10.1093/geronb/gbad150)
Supplement: gbad150_suppl_Supplementary_Tables_S1-S7_Figures_S1 [file gbad150_suppl_supplementary_tables_s1-s7_figures_s1.docx]

***The Journals of Gerontology, Series B: Psychological Sciences and Social Sciences* Supplementary Material: Testing Bidirectionality in Associations of Awareness of Age-Related Gains and Losses with Physical, Mental, and Cognitive Functioning Across One Year: The Role of Age.**

| **Supplementary Table 1.**  *Descriptive Statistics for the Overall Study Sample and Those Excluded from the Study Sample.* | | | | |
| --- | --- | --- | --- | --- |
|  | Subsample who Reported AARC in 2019 and 2020  (N = 5,354) | Subsample who Reported AARC only in 2019  (N = 4,056) | p-value | Eta-squared (η^2^) |
| Age; M (SD) | 66.14 (6.89) | 65.51 (7.23) | < .001 | .002 |
| Women, n (%) | 4,159 (77.7) | 3175 (78.3) | .488 |  |
| Married, n (%) | 3,982 (79.2) | 3,015 (74.3) | .699 |  |
| Missing, n | 329 | 235 |  |  |
| Education, n (%) |  |  |  |  |
| Secondary education | 716 (13.4) | 574 (14.2) |  |  |
| Post-secondary education | 635 (11.9) | 444 (11.0) |  |  |
| Vocational qualification | 1,053 (19.7) | 825 (20.3) |  |  |
| Undergraduate degree | 1,851 (34.6) | 1,301 (32.1) |  |  |
| Post-graduate degree | 912 (17.0) | 751 (18.5) |  |  |
| Doctorate | 187 (3.5) | 161 (4.0) |  |  |
| White ethnicity, n (%) | 5,282 (98.7) | 3,990 (98.4) | .558 |  |
| Employed, n (%) | 2,064 (39.5) | 1,842 (46.7) | < .001 |  |
| Missing, n | 129 | 108 |  |  |
| Functional difficulties, M (SD) | .16 (.76) | .17 (.78) | .4307 | .0001 |
| Anxiety, M (SD) | 8.40 (2.45) | 8.57 (2.59) | .002 | .001 |
| Missing, n | 43 | 101 |  |  |
| Depression, M (SD) | 11.34 (2.88) | 11.63 (3.14) | < .001 | .002 |
| Missing, n | 41 | 101 |  |  |
| Verbal reasoning, M (SD) | 38.09 (10.56) | 36.36 (10.39) | < .001 | .01 |
| Paired associate learning, M (SD) | 4.75 (.93) | 4.66 (.94) | < .001 | .002 |
| Self-ordered search, M (SD) | 7.81 (2.53) | 7.41 (2.79) | < .001 | .02 |
| Digit span, M (SD) | 7.65 (1.56) | 7.50 (1.48) | < .001 | .002 |
| *Note.* For eta-squared effect sizes between 0.01 and 0.05 are interpreted as small, between 0.06 and 0.13 are interpreted as moderate and of 0.14 or above are interpreted as large (Cohen, 1988). | | | | |

| **Supplementary Table 2.** *Baseline Descriptive Statistics for Study Participants by Age Groups.* | | | | | | |
| --- | --- | --- | --- | --- | --- | --- |
|  | Late Midlife  (N= 2,385) | | Early Old Age  (N= 2,430) | | Advanced Old Age  (N= 539) | |
|  | Baseline | One-year follow-up | Baseline | One-year follow-up | Baseline | One-year follow-up |
| Age; M (SD) | 59.97 (3.31) |  | 69.38 (2.76) |  | 78.78 (3.55) |  |
| Women, n (%) | 2,010 (84.3) |  | 1,808 (74.4) |  | 341 (63.3) |  |
| Married, n (%) | 1,919 (86.3) |  | 1,739 (76.0) |  | 324 (63.2) |  |
| Missing, n | 160 |  | 143 |  | 26 |  |
| Education, n (%) |  |  |  |  |  |  |
| Secondary education | 223 (9.4) |  | 378 (15.6) |  | 115 (21.3) |  |
| Post-secondary education | 294 (12.3) |  | 284 (11.7) |  | 57 (10.6) |  |
| Vocational qualification | 477 (20.0) |  | 482 (19.8) |  | 94 (17.4) |  |
| Undergraduate degree | 894 (37.5) |  | 792 (32.6) |  | 165 (30.6) |  |
| Post-graduate degree | 433 (18.2) |  | 405 (16.7) |  | 74 (13.7) |  |
| Doctorate | 64 (2.7) |  | 89 (3.7) |  | 34 (6.3) |  |
| White ethnicity, n (%) | 2,384 (98.5) |  | 2,399 (98.7) |  | 535 (99.3) |  |
| Employed, n (%) | 1,593 (70.1) |  | 437 (18.1) |  | 34 (6.3) |  |
| Missing, n | 112 |  | 17 |  | 0 |  |
| Awareness of age-related gains, M (SD) | 18.04 (3.98) | 18.26 (3.81) | 17.77 (3.84) | 18.37 (3.62) | 17.38 (3.76) | 18.00 (3.74) |
| Awareness of age-related losses, M (SD) | 9.23 (3.08) | 9.48 (3.19) | 9.95 (3.09) | 10.37 (3.22) | 11.75 (3.49) | 12.41 (3.59) |
| Functional difficulties, M (SD) | .15 (.83) | .17 (.82) | .14 (.65) | .16 (.65) | .28 (.88) | .47 (1.24) |
| Missing, n | 0 | 671 | 0 | 658 | 0 | 150 |
| Depression, M (SD) | 11.67 (3.15) | 11.51 (3.25) | 11.08 (2.60) | 11.05 (2.73) | 11.10 (2.67) | 11.58 (3.19) |
| Missing, n | 14 | 836 | 22 | 890 | 5 | 201 |
| Anxiety; M (SD) | 8.68 (2.63) | 8.67 (2.77) | 8.20 (2.31) | 8.28 (2.41) | 8.08 (2.01) | 8.35 (2.44) |
| Missing, n | 15 | 837 | 23 | 889 | 5 | 201 |
| Verbal reasoning, M (SD) | 40.36 (10.79) | 42.12 (11.33) | 37.35 (9.82) | 37.85 (10.47) | 31.98 (9.81) | 32.81 (9.38) |
| Missing, n | 688 | 610 | 614 | 641 | 132 | 151 |
| Paired associate learning, M (SD) | 4.90 (.93) | 4.29 (.97) | 4.71 (.91) | 4.00 (.98) | 4.34 (0.91) | 3.70 (0.96) |
| Missing, n | 688 | 597 | 614 | 626 | 132 | 148 |
| Self-ordered search, M (SD) | 8.22 (2.40) | 8.15 (1.98) | 7.65 (2.54) | 7.65 (1.84) | 6.83 (2.61) | 7.16 (1.76) |
| Missing, n | 688 | 564 | 614 | 584 | 132 | 133 |
| Digit span, M (SD) | 7.80 (1.52) | 7.89 (1.75) | 7.63 (1.55) | 7.55 (1.65) | 7.16 (1.63) | 7.03 (2.04) |
| Missing, n | 688 | 629 | 614 | 584 | 132 | 161 |
| Self-reported diagnosis of mild cognitive impairment | 6 (0.3) |  | 12 (0.5) |  | 5 (0.9) |  |

*Note.* Overall study sample N = 5,354.

| **Supplementary Table 3.** *Bidirectional Associations of AARC-Gains and AARC-Losses With Functional Difficulties by Age Group* | | | | | | | | |
| --- | --- | --- | --- | --- | --- | --- | --- | --- |
| **Age Group** | **AARC-Gains** | | | | **AARC-Losses** | | | |
|  | Outcomes at follow-up | Predictors at baseline | β (95% CI) | p-value | Outcomes at follow-up | Predictors at baseline | β (95% CI) | p-value |
| *Late Midlife – Unadjusted models (N= 1,714)* |  |  |  |  |  |  |  |  |
|  | AARC-gains | AARC-gains | .61 (.59; .63) | < .001 | AARC-losses | AARC-losses | .72 (.70; .74) | < .001 |
|  |  | Functional difficulties | .01 (-.03; .04) | .766 |  | Functional difficulties | .05 (.03; .08) | < .001 |
|  | Functional difficulties | AARC-gains | -.02 (-.06; .02) | .312 | Functional difficulties | AARC-losses | .22 (.18; .26) | < .001 |
|  |  | Functional difficulties | .53 (.50; .56) | < .001 |  | Functional difficulties | .48 (.45; .51) | < .001 |
| *Young- Old Age – Unadjusted models (N= 1,772)* | AARC-gains | AARC-gains | .61 (.58; .63) | < .001 | AARC-losses | AARC-losses | .72 (.70; .74) | < .001 |
|  |  | Functional difficulties | -.02 (-.06; .01) | .121 |  | Functional difficulties | .05 (.02; .07) | .005 |
|  | Functional difficulties | AARC-gains | -.01 (-.05; .03) | .593 | Functional difficulties | AARC-losses | .15 (.11; .19) | < .001 |
|  |  | Functional difficulties | .46 (.42; .49) | < .001 |  | Functional difficulties | .43 (.39; .47) | < .001 |
| *Old-Old Age- Unadjusted models (N= 382)* | AARC-gains | AARC-gains | .64 (.59; .68) | <.001 | AARC-losses | AARC-losses | .70 (.66; .74) | <.001 |
|  |  | Functional difficulties | -.02 (-.08; .05) | .636 |  | Functional difficulties | .05 (-.01; .11) | .016 |
|  | Functional difficulties | AARC-gains | -.06 (-.15; .04) | .238 | Functional difficulties | AARC-losses | .31 (.23; .40) | <.001 |
|  |  | Functional difficulties | .37 (.28; .46) | <.001 |  | Functional difficulties | .30 (.20; .39) | <.001 |

| **Supplementary Table 4.** *Bidirectional Associations of AARC-Gains and AARC-Losses With Anxiety by Age Group* | | | | | | | | |
| --- | --- | --- | --- | --- | --- | --- | --- | --- |
| **Age Group** | **AARC-Gains** | | | | **AARC-Losses** | | | |
|  | Outcomes at follow-up | Predictors at baseline | β (95% CI) | p-value | Outcomes at follow-up | Predictors at baseline | β (95% CI) | p-value |
| *Late Midlife – Unadjusted models (N= 1,536)* | AARC-gains | AARC-gains | .61 (.58; .63) | < .001 | AARC-losses | AARC-losses | .71 (.69; .73) | < .001 |
|  |  | Anxiety | -.01 (-.04; .02) | .454 |  | Anxiety | .06 (.03; .09) | < .001 |
|  | Anxiety | AARC-gains | .03 (-.01; .07) | .188 | Anxiety | AARC-losses | .20 (.16; .24) | < .001 |
|  |  | Anxiety | .52 (.48; .55) | < .001 |  | Anxiety | .46 (.43; .50) | < .001 |
| *Young-Old Age- Unadjusted models (N= 1,518)* | AARC-gains | AARC-gains | .61 (.58; .63) | < .001 | AARC-losses | AARC-losses | .71 (.69; .73) | < .001 |
|  |  | Anxiety | .01 (-.03; .04) | .711 |  | Anxiety | .09 (.07; .12) | < .001 |
|  | Anxiety | AARC-gains | -.01 (-.05; .04) | .835 | Anxiety | AARC-losses | .17 (.12; .21) | < .001 |
|  |  | Anxiety | .47 (.44; .51) | < .001 |  | Anxiety | .43 (.39; .47) | < .001 |
| *Old-Old Age – Unadjusted models (N = 333)* | AARC-gains | AARC-gains | .64 (.59; .69) | <.001 | AARC-losses | AARC-losses | .70 (.66; .75) | <.001 |
|  |  | Anxiety | -.03 (-.09; .04) | .389 |  | Anxiety | .02 (-.04; .08) | .490 |
|  | Anxiety | AARC-gains | -.02 (-.11; .07) | .659 | Anxiety | AARC-losses | .14 (.05; .23) | .002 |
|  |  | Anxiety | .59 (.52; .66) | <.001 |  | Anxiety | .55 (.48; .63) | <.001 |

| **Supplementary Table 5.**  *Bidirectional Associations of AARC-Gains and AARC-Losses With Depression by Age Group* | | | | | | | | |
| --- | --- | --- | --- | --- | --- | --- | --- | --- |
| **Age Group** | **AARC-Gains** | | | | **AARC-Losses** | | | |
|  | Outcomes at follow-up | Predictors at baseline | β (95% CI) | p-value | Outcomes at follow-up | Predictors at baseline | β (95% CI) | p-value |
| *Late Midlife – Unadjusted models (N= 1,538)* | AARC-gains | AARC-gains | .61 (.58; .63) | < .001 | AARC-losses | AARC-losses | .69 (.67; .72) | < .001 |
|  |  | Depression | -.02 (-.05; .01) | .229 |  | Depression | .10 (.07; .13) | < .001 |
|  | Depression | AARC-gains | -.0001 (-.04; .04) | .997 | Depression | AARC-losses | .27 (.22; .31) | < .001 |
|  |  | Depression | .55 (.52; .58) | < .001 |  | Depression | .45 (.42; .49) | < .001 |
| *Young-Old Age – Unadjusted models (1,519)* | AARC-gains | AARC-gains | .61 (.58; .63) | < .001 | AARC-losses | AARC-losses | .69 (.67; .71) | < .001 |
|  |  | Depression | -.01 (-.04; .02) | .587 |  | Depression | .12 (.09; .15) | < .001 |
|  | Depression | AARC-gains | -.02 (-.06; .02) | .388 | Depression | AARC-losses | .21 (.17; .26) | < .001 |
|  |  | Depression | .57 (.54; .60) | < .001 |  | Depression | .50 (.47; .54) | < .001 |
| *Old-Old Age- Unadjusted models (N= 333)* | AARC-gains | AARC-gains | .63 (.58; .68) | <.001 | AARC-losses | AARC-losses | .68 (.64; .73) | <.001 |
|  |  | Depression | -.05 (-.12; .01) | .105 |  | Depression | .07 (.01; .13) | .029 |
|  | Depression | AARC-gains | -.05 (-.12; .03) | .234 | Depression | AARC-losses | .11 (.03; .19) | .007 |
|  |  | Depression | .70 (.65; .75) | <.001 |  | Depression | .66 (.60; .72) | <.001 |

| **Supplementary Table 6.**  *Bidirectional Associations of AARC-Gains and AARC-Losses With Scores on Verbal Reasoning by Age Group* | | | | | | | | |
| --- | --- | --- | --- | --- | --- | --- | --- | --- |
| **Age Group** | **AARC-Gains** | | | | **AARC-Losses** | | | |
|  | Outcomes at follow-up | Predictors at baseline | β (95% CI) | p-value | Outcomes at follow-up | Predictors at baseline | β (95% CI) | p-value |
| *Late Midlife – Unadjusted models (N= 1,229)* | AARC-gains | AARC-gains | .61 (.58; .63) | < .001 | AARC-losses | AARC-losses | .73 (.71; .75) | < .001 |
|  |  | Verbal reasoning | -.02 (-.06; .01) | .206 |  | Verbal reasoning | -.02 (-.05; .01) | .109 |
|  | Verbal reasoning | AARC-gains | -.03 (-.07; .0001) | .051 | Verbal reasoning | AARC-losses | -.003 (-.04; .03) | .882 |
|  |  | Verbal reasoning | .77 (.75; .79) | < .001 |  | Verbal reasoning | .77 (.75; .79) | < .001 |
| *Young-Old Age- Unadjusted models (N= 1,305)* | AARC-gains | AARC-gains | .61 (.58; .63) | < .001 | AARC-losses | AARC-losses | .73 (.71; .75) | < .001 |
|  |  | Verbal reasoning | -.02 (-.05; .02) | .276 |  | Verbal reasoning | -.04 (-.07; -.01) | .007 |
|  | Verbal reasoning | AARC-gains | -.011 (-.03; .03) | .946 | Verbal reasoning | AARC-losses | -.04 (-.07; -.01) | .044 |
|  |  | Verbal reasoning | .78 (.76; .80) | < .001 |  | Verbal reasoning | .78 (.76; .80) | < .001 |
| *Old-Old Age – Unadjusted models (N= 286)* | AARC-gains | AARC-gains | .64 (.58; .69) | <.001 | AARC-losses | AARC-losses | .71 (.67; .75) | <.001 |
|  |  | Verbal reasoning | -.03 (-.10; .04) | .457 |  | Verbal reasoning | .02 (-.04; .09) | .461 |
|  | Verbal reasoning | AARC-gains | -.01 (-.08; .06) | .738 | Verbal reasoning | AARC-losses | -.10 (-.17; -.02) | .009 |
|  |  | Verbal reasoning | .77 (.73; .82) | <.001 |  | Verbal reasoning | .77 (.72; .81) | <.001 |

| **Supplementary Table 7.** *Bidirectional Associations of AARC-Gains and AARC-Losses With Scores on Working Memory in* | | | | | | | | |
| --- | --- | --- | --- | --- | --- | --- | --- | --- |
| **Age Group** | **AARC-Gains** | | | | **AARC-Losses** | | | |
|  | Outcomes at follow-up | Predictors at baseline | β (95% CI) | p-value | Outcomes at follow-up | Predictors at baseline | β (95% CI) | p-value |
| *Late Midlife – Unadjusted models (N= 1,229)* | AARC-gains | AARC-gains | .61 (.58; .63) | <.001 | AARC-losses | AARC-losses | .73 (.71; .75) | <.001 |
|  |  | Working memory | -.05 (-.08; -.01) | .015 |  | Working memory | -.02 (-.05; .01) | .170 |
|  | Working memory | AARC-gains | -.03 (-.07; .01) | .118 | Working memory | AARC-losses | -.02 (-.06; .02) | .412 |
|  |  | Working memory | .57 (.54; .61) | <.001 |  | Working memory | .57 (.54; .61) | <.001 |
| *Young-Old Age – Unadjusted models (N= 1,305)* | AARC-gains | AARC-gains | .61 (.58; .63) | <.001 | AARC-losses | AARC-losses | .73 (.71; .75) | <.001 |
|  |  | Working memory | .01 (-.03; .04) | .715 |  | Working memory | -.06 (-.09; -.03) | <.001 |
|  | Working memory | AARC-gains | -.05 (-.09; -.01) | .025 | Working memory | AARC-losses | -.04 (-.08; .004) | .081 |
|  |  | Working memory | .57 (.53; .61) | <.001 |  | Working memory | .57 (.54; .61) | <.001 |
| *Old-Old Age – Unadjusted models (N= 286)* | AARC-gains | AARC-gains | .64 (.59; .69) | <.001 | AARC-losses | AARC-losses | .71 (.67; .75) | <.001 |
|  |  | Working memory | -.02 (-.10; .05) | .515 |  | Working memory | -.01 (-.98; .06) | .846 |
|  | Working memory | AARC-gains | -.03 (-.12; .06) | .502 | Working memory | AARC-losses | -.02 (-.11; .07) | .680 |
|  |  | Working memory | .56 (.48; .64) | <.001 |  | Working memory | .56 (.48; .64) | <.001 |

**Supplementary Figure 1.**

**
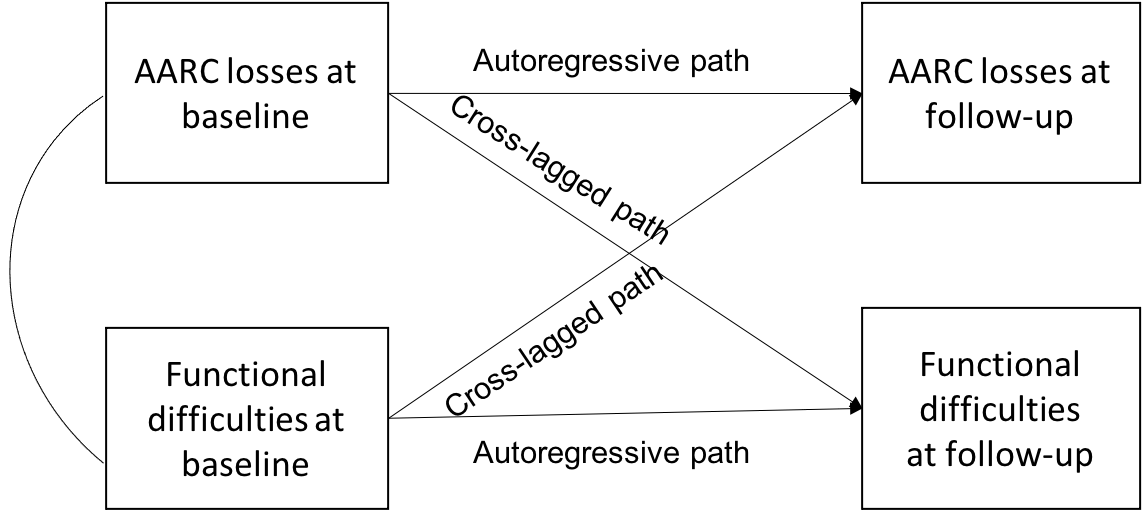
**
